# Regulatory Scientific Advice on Non-Inferiority Drug Trials

**DOI:** 10.1371/journal.pone.0074818

**Published:** 2013-09-05

**Authors:** Grace Wangge, Michelle Putzeist, Mirjam J. Knol, Olaf H. Klungel, Christine C. Gispen-De Wied, Antonius de Boer, Arno W. Hoes, Hubert G. Leufkens, Aukje K. Mantel-Teeuwisse

**Affiliations:** 1 Division of Pharmacoepidemiology and Clinical Pharmacology, Utrecht Institute for Pharmaceutical Sciences (UIPS), Utrecht University, Utrecht, The Netherlands; 2 Medicines Evaluation Board, Utrecht, The Netherlands; 3 Rijksinstituut voor Volksgezondheid en Milieu (RIVM), National Institute for Public Health and the Environment, Centre for Infectious Disease Control Netherlands (CIb), Epidemiology and Surveillance Unit (EPI), Bilthoven, The Netherlands; 4 Julius Centre for Health Sciences and Primary Care, University Medical Centre Utrecht, Utrecht, The Netherlands; University of British Columbia, Canada

## Abstract

The active-controlled trial with a non-inferiority design has gained popularity in recent years. However, non-inferiority trials present some methodological challenges, especially in determining the non-inferiority margin. Regulatory guidelines provide some general statements on how a non-inferiority trial should be conducted. Moreover, in a scientific advice procedure, regulators give companies the opportunity to discuss critical trial issues prior to the start of the trial. The aim of this study was to identify potential issues that may benefit from more explicit guidance by regulators. To achieve this, we collected and analyzed questions about non-inferiority trials posed by applicants for scientific advice in Europe in 2008 and 2009, as well as the responses given by the European Medicines Agency (EMA). In our analysis we included 156 final letters of advice from 2008 and 2009, addressed to 94 different applicants (manufacturers). Our analysis yielded two major findings: (1) applicants frequently asked questions ‘whether’ and ‘how’ to conduct a non-inferiority trial, 26% and 74%, respectively, and (2) the EMA regulators seem mainly concerned about the choice of the non-inferiority margin in non-inferiority trials (36% of total regulatory answers). In 40% of the answers, the EMA recommended using a stricter margin, and in 10% of the answers regarding non-inferiority margins, the EMA questioned the justification of the proposed non-inferiority margin.

We conclude that there are still difficulties in selecting the appropriate methodology for non-inferiority trials. Straightforward and harmonized guidance regarding non-inferiority trials is required, for example on whether it is necessary to conduct such a trial and how the non-inferiority margin is determined. It is unlikely that regulatory guidelines can cover all therapeutic areas; therefore, in some cases regulatory scientific advice may be used as an opportunity for tailored advice.

## Introduction

Randomized placebo-controlled trials (RCTs) are considered the gold standard to confirm the efficacy of a drug. Currently, active-controlled trials are often performed instead of or in addition to placebo-controlled trials as the basis for marketing authorization and reimbursement decisions. A previous study showed that for 48% of all new medicines approved between 1999 and 2005, at least one active-controlled trial had been conducted during the development phase [1].

An active-controlled trial may have a non-inferiority design. A non-inferiority trial intends to demonstrate that the new drug is not worse than its comparator (a drug previously shown to be more effective than a placebo) to a certain limit (the non-inferiority margin), thus indirectly showing that the new treatment is effective (i.e. more effective than a placebo). However, non-inferiority trials pose several methodological challenges, especially in determining the non-inferiority margin. Previously we found that in 22% of the non-inferiority trials, the choice of the non-inferiority margin was based merely on assumptions made by the investigators. [2].

The International Conference on Harmonization of Technical Requirements for Registration of Pharmaceuticals for Human Use (ICH) E9 [3], the ICH E10 [4], the European Medicines Agency (EMA) guidelines [5], [6] and the US Food and Drug Administration (FDA) draft guideline on non-inferiority trials [7] are the guidelines currently available that advise on the appropriate set-up of non-inferiority trials. Most of these guidelines only have general information on how to conduct such trials. However, in their guidelines for trials in certain therapeutic areas (such as diabetes mellitus and infectious diseases), the FDA and EMA provide more explicit guidance on how to apply a non-inferiority trial methodology. [8], [9] Interestingly, in the guidelines that present a specific non-inferiority margin, there are discrepancies between the FDA and EMA. For example, in the 2008 draft FDA guidance for diabetes mellitus, a non-inferiority margin in HbA1C reduction is suggested of 0.3% or 0.4%, while the 2011 EMA guideline suggests a non-inferiority margin of 0.3%. [8] A difference of 0.1% of HbA1C may not be significant in the clinic, but this difference will result in two largely different sample size calculations in non-inferiority trials.

Apart from guidelines, regulators provide the opportunity for companies to discuss critical trial issues prior to the start of the trial, to improve the quality of pre-registration trials. Scientific advice is an important part of such discussions. In Europe, scientific advice can be sought either from the EMA or from one or more national regulatory agencies. [10] Regulatory scientific advice can be asked as often as deemed necessary by an applicant, who is not obliged to adhere to the advice received or committed to accept any result of a scientific advice procedure. In a previous study, we found that one of the top five questions posed by the applicants during a scientific advice procedure was on study design. [11] However, the questions and responses specifically related to the non-inferiority design were not assessed in detail. Therefore, it remained largely unknown whether companies often ask questions specifically related to the non-inferiority trial design, and what their questions are and what replies they receive. In our previous review, we found that most non-inferiority trials were financed by the pharmaceutical industry (73.7%). [12] In this study, we identified questions on non-inferiority trials that were posed by applicants for scientific advice in Europe in 2008 and 2009, and the responses given by the EMA. Our analysis of the questions about non-inferiority trials posed by applicants in scientific advice dialogues with the EMA could identify any complex issues in the regulation of non-inferiority trials that may benefit from more explicit regulatory guidance.

## Methods

To identify which documents discussed non-inferiority trials, we used the keyword ‘inferior’ to search in the Dutch Medicines Evaluation Board (MEB) database among final letters of advice from 2008–2009 from the EMA, represented by the Committee for Medicinal Products for Human use (CHMP). This scientific advice is confidential and is therefore not publicly available; to gain access to the data, researchers must be affiliated with the EMA/MEB. Four authors (GW, MP, CGDW and HL) are affiliated with the MEB and a confidentiality agreement was signed between the parties involved (Utrecht University and MEB) prior to the start of the study. At the time of our study, scientific advice from more recent years than 2008 and 2009 was not fully available.

Each final letter of advice consisted of questions from the applicants (company questions), followed by an elaboration of the particular question, the company position, and the CHMP response to each question. We excluded documents and company questions that did not discuss non-inferiority trials on efficacy or that discussed bioequivalence trials.

The following information was collected for each scientific advice briefing document: whether it was a follow-up to a previous scientific advice application, whether the drug was classified as an orphan drug, and what the (description) therapeutic target group of the drug was. The drugs were categorized by their therapeutic target group according to their first level of Anatomical Therapeutic Chemical (ATC) classification. [13] If an ATC classification was missing, the anatomical main group was determined based on the intended indication of the product.

Each company question, accompanying company position and CHMP response was scored according to the topic of interest. The topics of interest were divided into two types: ‘general’ and ‘specific’. General topics covered discussions about the strategic/overall development process of a drug. The specific topics consisted of topics unique to non-inferiority trials and topics unrelated to non-inferiority trials. Topics unique to non-inferiority trial included questions on whether or not a non-inferiority trial should be conducted (‘whether’ questions, related to the choice of a non-inferiority study design) and topics that discussed technical issues regarding how a non-inferiority trial should be conducted (‘how’ questions, related to the type of comparator, non-inferiority margin, non-inferiority sample size calculations, per-protocol or intention-to-treat analysis, and the possible switch from a non-inferiority design to a superiority design or vice versa). Topics that were not unique to non-inferiority trials discussed aspects of clinical trials that were not specific to non-inferiority trials, for example trial inclusion-exclusion criteria and types of endpoints (See Table 1 for further details and examples).

**Table 1:** Examples of topics related to non-inferiority trials in company questions, company positions and CHMP responses identified in final letters of advice.

Topics			Definition	Example			
				Company question	Company position		CHMP answer
Unique tonon-inferioritytrials	WHETHER a non-inferiority trialsshould be used		Discusses non-inferiority trial design	Does the agency supportthe selected**non-inferiority**trial design for thephase III study inpatients with disease Y?	An open-labelled,randomized, multi-centre,parallel group study willbe conducted in adultpatients for a period of 6–8months. The primary objectiveof this study is todemonstrate that ABC isjust as efficacious asDEF, the current therapy.		CHMP agrees with the non-inferiority design. However, a two-arm **non-inferiority** study is considered inadvisable due to concerns relating to assay-sensitivity. For these reasons, inclusion of a placebo control is strongly recommended in addition to the active control.
	HOW- topic	Type ofcomparator	a) Discusses the typeof comparator *or*b) The use of anadditional placeboarm in the trial	Does the agency agreewith the use of drugD as the active comparatorin this **non-inferiority**trial?	Drug D is recommendedby the Association X as thecurrent therapy of choicefor disease Y.		The choice of active comparator is justified.
		Non-inferioritymargin	Discusses the non-inferiority margin	Does the scientific adviceworking party consideran X% **non-inferiority**margin acceptable?	A minimum clinicalrelevant difference to bedetected is suggested to X%(**non-inferiority** margin),preserving 2/3 of theeffect of ABC vs.placebo on primary endpoint.		The CHMP agrees
		Non-inferioritydata analysis^$^	a) Discusses dataanalysis of a non-inferiority trial,including samplesize calculationfor non-inferioritytrial *or*b) Controlling typeI error innon-inferioritytrial	In this trial, the primaryanalysis is performedon the per-protocolpopulation (with asecondary analysisperformed on the intent-to-treat population),with a sample sizeof Z patients.Does CHMP agree?	Sample size was calculatedbased on a 5% betterresponse rate in the drug Darm, a **non-inferiority** marginof X%, a one-sided significancelevel 2.5%, and apower of 80%.		The CHMP disagrees. A sample size of X patients is necessary to exclude a significant activity difference. We recommend to do the primary analysis in both per-protocol and intention-to-treat analysis.
		Per-protocolor intention-to-treat ^$^	Discusses the choiceof per-protocol orintention-to-treatanalysis	*See example above*			
		Switching	Discusses a plan toswitch fromnon-inferiority tosuperiority or fromsuperiority tonon-inferiority	Is the test procedure- testing first for**non inferiority** followed bysuperiority – acceptable?	The sponsor believes thatthe described procedureis in line with EMEA/CHMPguidelines forswitching betweensuperiority and **non-** **inferiority.**	Switching from **non-inferiority** to superiority as proposed is acceptable.	
Not unique to non-inferiority trials			Discusses topics relatedto conduct of the trial,that are not specificallyrelated tonon-inferiority trials	We propose modifying theprimary compositeendpoint to includehospitalization in this**non-inferiority** trial.	The proposal to exchangethe two compositeendpoints would addressthe lower than anticipatedevent rate and would stillallow the assessment ofthe impact of ABC on acomposite endpoint.	There is reluctance to agree to the proposal, based on clinical grounds. Adding hospitalization would add a component that has a different relevance than the components that were originally agreed on as making a valid composite primary endpoint.	
General			Discusses ‘Strategyquestion’	Does the Agency agreethat a single phase III studywith the proposed primaryendpoint and statisticalevaluation will providesufficient data forapproval?	Since, at this stage, noapproved drug is availablefor patients with disease Y,it is considered acceptableto base potential approval ofdrug D for this disease on theproposedphase III program.	Approval of drug D based on the current program will be possible. A new agent or indication should have a safety/efficacy profile **non-inferior** to marketed comparators.	

Note: $ An example that multiple topics can be discussed in one company question and the related company position.

To ensure confidentiality, the data presented in the table are not the original sentences found in the scientific advice applications.

In each company question, company position or CHMP response, multiple topics may be discussed, and all these topics were included separately in the analyses. Additional topics that were found in the CHMP response but not in the accompanying company question or position were classified as ‘extra information’. In addition the results were presented for different therapeutic target groups separately.

Author GW searched and extracted all company questions, company positions and CHMP answers in the scientific advice documents, while the classification was carried out by both GW and MP separately. In case of discrepancies (n = 5), GW and MP tried to reach agreement on the classification first, before consulting AM-T and MK to reach final consensus. Subsequently, data were analyzed by GW and MP in a descriptive way. In addition, they assessed the topics according to their therapeutic target group.

## Results

### Search Result and General Characteristics

**Figure 1:**
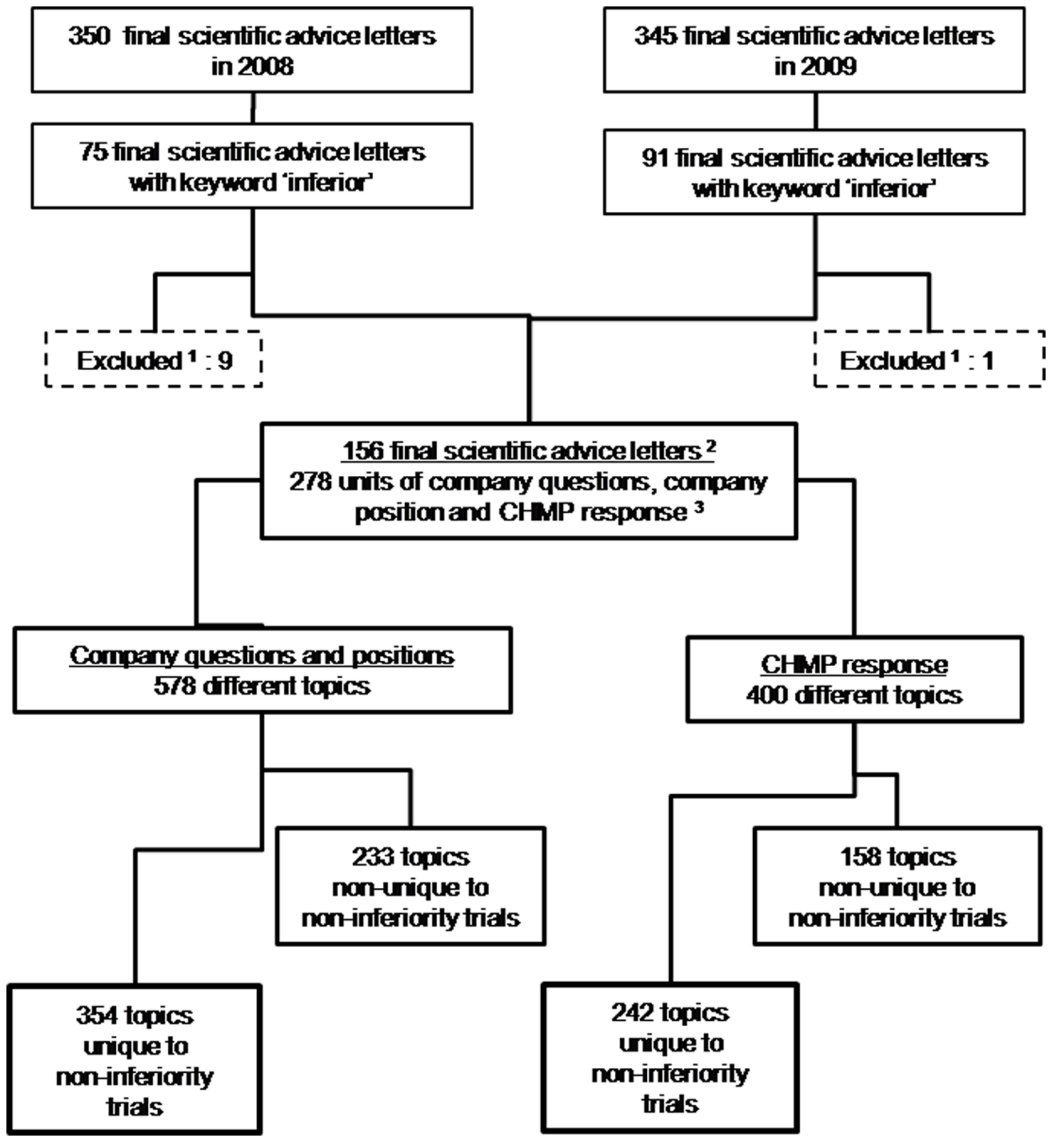
Search strategy and result. Note: 1. Final scientific advice letters were excluded if they were not related to non-inferiority trials; 2. In the analysis, 156 final scientific advice letters were included, containing 278 different questions (including their company position and CHMP response); 3. Each company question, company position and CHMP response may contain more than one topic.

In total 75 documents from 2008 (22% of the 350 final advice documents from 2008) and 91 documents from 2009 (26% of the 345 documents from 2009) contained the keyword ‘inferior’. We excluded nine documents from 2008 and one document from 2009, because they were not related to non-inferiority trials. Finally, 156 documents were included in our analysis: 66 final advice letters from 2008 and 90 final advice letters from 2009. These 156 final advice letters had been addressed to 94 different applicants (manufacturers). In total, the documents contained 278 company questions, company positions and CHMP responses related to non-inferiority trials (Figure 1).

Characteristics of the documents and questions are presented in Table 2. Of the therapeutic groups, antineoplastic and immunomodulating products were discussed most often (22% of the final advice letters included), followed by alimentary tract and metabolism products (17%) and anti-infectives (16%).

**Table 2:** General overview of scientific advice applications.

	Eligible final letter of adviceN = 156 (%)	Company questions^1^N = 278 (%)
Follow-up application	36 (23)	51 (18)
Orphan drugs	14 (9)	23 (8)
Therapeutic target group^2^		
Antineoplastic and immunomodulating products	34 (22)	63 (23)
Alimentary tract and metabolism	27 (17)	48 (17)
Anti-infective drugs	25 (16)	47 (17)
Blood and blood-forming organs	15 (10)	31 (11)
Respiratory system	10 (6)	19 (7)
Musculoskeletal system	13 (8)	16 (6)
Systemic hormonal preparations, excluding sex hormones and insulin	7 (4)	14 (5)
Nervous system	7 (4)	10 (4)
Others	18 (12)	30 (11)

Note: 1.Each final letter of advice may consist of more than one company question; 2.Therapeutic target group based on 1^st^ level of Anatomic Therapeutic Chemical (ATC) codes.

### Topics of Discussion

Within the 278 company questions and positions related to non-inferiority trials, a total of 587 different topics were discussed. Of these, 101 were classified as general topics, asking advice regarding the overall development strategy, which may include a non-inferiority RCT. Issues that were more specific but not unique to non-inferiority trial design were identified 132 times. The remaining 354 topics were unique to non-inferiority trials. In the CHMP responses, a total of 400 different topics were discussed. Of these, 242 topics were unique to non-inferiority trials.

Among the topics unique to non-inferiority trials, both topics of ‘whether’ and ‘how’ to conduct a non-inferiority trial frequently appeared in the company questions, company positions and CHMP responses, but ‘how’ questions were asked more frequently than ‘whether’ questions: the ‘how’ questions represented 74% of total non-inferiority topics asked and 72% of the total CHMP responses, whereas the ‘whether’ questions made up the remaining 26% of total topics asked and 28% of the total CHMP non-inferiority unique responses.

Among the ‘how’ topics, the non-inferiority margin was most frequently discussed: 98 of the 354 company questions and positions (28%) and 86 of the 242 CHMP-responses of all the topics unique to non-inferiority trials (36%). In 42 of the 86 CHMP responses that discussed non-inferiority margin (49%), the CHMP supported the applicants’ non-inferiority margin proposal, while in another 35 responses (41%), CHMP recommended a stricter margin. In the remaining 9 CHMP responses (10%), the justification of the margin was questioned by the CHMP, but no specific advice on its magnitude was given.

The topic of data analysis (sample size calculation and type I error) was the second most frequently discussed topic. This topic was discussed in 87 (25%) of the company questions and positions and in 50 (21%) of the CHMP-responses related to topics unique to non-inferiority trials. In 48 of the 50 CHMP responses that discussed a non-inferiority data analysis (96%), the CHMP supported the applicants’ data analysis plan, while in one response, the CHMP recommended that the applicant revised the alpha level and in another response, the CHMP suggested that the applicant came up with a new plan for data-interim analysis.

The topic of switching between a superiority and a non-inferiority design appeared least often in company questions and positions (6% of the total topics asked), and the topic of ‘per-protocol or intention-to-treat appeared least often in CHMP responses (3% of the total CHMP responses; see Table 3).

**Table 3:** Frequency of topics unique to non-inferiority trials occurring in company questions and company positions or in CHMP responses based on therapeutic target group.

Topic		All therapeuticgroups		Antineoplastic andimmunomodulatingdrugs		Alimentarytractandmetabolismdrugs		Anti-infectivedrugs		Other drugs	
		N (% of total)		N (% of total)		N (% of total)		N (% of total)		N (% of total)	
		Company questionsandcompanypositions	CHMP responses	Company questions and company positions	CHMP responses	Companyquestionsandcompanypositions	CHMP responses	Companyquestionsandcompany positions	CHMP responses	Companyquestionsandcompanypositions	CHMP responses
Whether non-inferiority trials should be used		92 (26)	67 (28)	21 (25)	11 (20)	11 (22)	10 (26)	4 (20)	3 (21)	56 (28)	43 (32)
HOW - topics	Type of comparator	34 (10)	22 (9)	8 (9)	5 (9)	4 (8)	6 (15)	4 (20)	1 (7)	18 (9)	10 (7)
	Non-inferiority margin	98 (28)	86 (36)	21 (25)	21 (38)	15 (31)	11 (28)	8 (40)	8 (57)	54 (27)	46 (34)
	Non-inferiority data analysis	87 (25)	50 (21)	25 (29)	15 (27)	15 (31)	10 (26)	3 (15)	2 (14)	44 (22)	23 (17)
	Per-protocol or intention-to-treat analysis	22 (6)	8 (3)	5 (6)	0 (0)	1 (2)	0 (0)	0 (0)	0 (0)	16 (8)	8 (6)
	Switching	21 (6)	9 (4)	5 (6)	3 (5)	3 (6)	2 (5)	1 (5)	0 (0)	12 (6)	4 (3)
TOTAL		354 (100)	242 (100)	85 (100)	55 (100)	49 (100)	39 (100)	20 (100)	14 (100)	200 (100)	134 (100)

In addition, Table 3 shows the differences and similarities in company questions, company positions and CHMP responses between the three therapeutic target groups discussed most often. For antineoplastic and immunomodulating products and for alimentary tract and metabolism products, the topics of data analysis mostly appeared in company questions and company positions (29% and 31%, respectively), while for anti-infective drugs the non-inferiority margin was discussed most often (40% of total topics asked within the therapeutic area). Most company questions and positions relating to other drugs dealt with the non-inferiority study design (28% of the total topics asked).

Among the CHMP responses, the non-inferiority margin was the topic that appeared most often in all three therapeutic target groups (38% of the total CHMP responses in antineoplastic and immunomodulating drugs, 28% in alimentary and metabolism drugs and 57% in anti-infective drugs).The non-inferiority margin was also discussed most often in the CHMP responses for other drugs (35% of the total CHMP responses).

‘Extra information’, i.e. unsolicited answers given by the CHMP, pertained to ‘how’ to perform a non-inferiority study more often than to ‘whether’ to perform a non-inferiority trial (data not shown). In alimentary tract and metabolism products, extra information was mostly given about ‘whether’ to perform a non-inferiority trial.

## Discussion

Our content analysis of 2008 and 2009 scientific advice on non-inferiority trials provided by the EMA shows that applicants frequently ask questions about ‘whether’ and ‘how’ to conduct such trials. In addition, the EMA’s main concern seems to be the non-inferiority margin applied in non-inferiority trials.

Interestingly, more than 25% of the questions were ’whether’ questions, and thus it seemed that applicants frequently have doubts about the need for a non-inferiority trial. This result illustrates that more explicit guidance is necessary on fundamental issues in non-inferiority trials, such as in what situations a non-inferiority trial could or should be applied. However, we realize that one general guideline may not be feasible for all therapeutic areas, for example in cases where the efficacy of the current standard therapy against placebo has not yet been fully established (e.g. anti-depressants). [14].

Our second finding shows that non-inferiority margins and data analysis were the specific topics discussed most frequently. This finding applied to all therapeutic areas. Moreover, in 40% of the CHMP responses on non-inferiority margins, a stricter margin was recommended. This concern was previously acknowledged by European regulators. [15], [16] Although the data analysis was often discussed by applicants, it seems that the CHMP did not regard this area as problematic. The CHMP mostly supported the applicants’ data-analysis proposal or had no additional remarks. A similar finding was also found for the topic of per-protocol and intention-to-treat analysis. This may have been the result of the straightforward nature of the data analysis and the clear guidelines. [3], [5] However, the large proportion of ‘how’ questions confirms that the methodology of non-inferiority trials is not straightforward, in particular the determination of the non-inferiority margin. [2] These facts underline the need for additional guidance for the applicants on technical issues, such as given previously by the EMA guidance [6] and draft FDA guidelines on non-inferiority trials. [7].

Our subgroup analysis showed that the non-inferiority trial design for alimentary tract and metabolism products (including diabetes products) is of specific concern to the CHMP since the CHMP often recommends a non-inferiority design for these products, even if the applicant does not ask for guidance on this subject. Apparently, the use of non-inferiority trials to confirm drug efficacy is still complex in this therapeutic area. The CHMP released their revised guidance on anti-diabetic drugs in 2011. [8] Besides superiority trials, this guidance recommends the use of non-inferiority trials in diabetes patients. This may help to clarify in which cases non-inferiority trials should be performed.

The 2011 guidance on anti-diabetic drugs described above included the recommendation of a non-inferiority margin of 0.3% HbA1C. A similar specific requirement was previously proposed by the EMA for anti-infective drugs, recommending a specific value of the non-inferiority margin (10%). [17] Although the numbers are small, we found that the specific requirements still resulted in questions on the non-inferiority margin in anti-infective drugs. Recently, in a version updated in 2011, the value of 10% was replaced by a general statement in the guideline on how to determine a non-inferiority margin. [18] This approach is in line with the draft FDA guideline 2010 [7], which recommends determining a non-inferiority margin based on historical data instead of using a single fixed value as margin. It remains to be seen whether this new approach will lead to a reduction or an increase in scientific advice questions related to non-inferiority trials. In the meantime, it is essential that regulators are aware of the difficulties faced by applicants, and scientific dialogue between both parties can support the regulators in improving guidance on non-inferiority trials. This study did not assess the clinical impact of different scientific recommendations for non-inferiority studies by regulatory authorities on public health, but this is an interesting topic that should be explored in a follow-up study. In a previous study, we conducted a survey among clinical experts and demonstrated that assessing an appropriate non-inferiority margin with an acceptable clinical impact is not a straightforward task, even for clinical experts. One of the reasons may lie in the difficulty to understand the non-inferiority concept and in the need for a better structured method. A survey and a Delphi method may work, but further research is required. [19].

Non-inferiority trials are considered controversial [20] because they provide the opportunity for patients to receive medicine that provides no extra benefit but could be less safe. Indeed, in a previous review we showed that the ethical concerns regarding non-inferiority trials are mainly related to exposing patients to drugs without the intention of showing that these drugs have any additional benefits. [21] However, even if showing superiority of an outcome is considered to be an integrated part of all trials, a non-inferiority aim for efficacy outcomes could be important and necessary. Patients may respond differently to medicinal products, and therefore it may be beneficial for patients to have more therapeutic choices on the market that are equally effective. In addition, marketing approval decisions will not be based solely on the result of one trial or on a single non-inferiority trial. The needs for transparency about the conduct of non-inferiority studies, including insight into the questions posed by sponsors conducting non-inferiority trials and into the interpretation of the results are essential for good non-inferiority trials practice.

The limitations of this study also need to be addressed. Firstly, our data came from final scientific advice documents, which did not give detailed information on the reasoning behind the advice given. To discuss best practices and understand choices in non-inferiority trials, analyses of scientific advice questions for specific therapeutic drug groups are needed, supplemented by a more in-depth qualitative study. Secondly, the advice documents contained only a few (statistical) details on the non-inferiority trials. The type of study was not always clear, for example whether it was an explorative study or a pivotal confirmative phase III study. In the final scientific advice documents, the process of determining the non-inferiority margin was mostly described in general terms. Thus, it was difficult to analyze the statistical reasoning behind the proposed non-inferiority margin. However, the questions in the scientific advice documents show the potential problems in non-inferiority trials faced by the applicants.

We conclude that difficulties still exist in selecting the appropriate methodology of non-inferiority trials. Straightforward and harmonized guidance on non-inferiority trials is needed, such as in which cases these trials need to be conducted and how the non-inferiority margin is to be determined. It is unlikely that regulatory guidelines (either as one general guideline or special sections on non-inferiority trials in disease-specific guidelines) can cover all therapeutic areas; therefore, in some cases regulatory scientific advice may be used as an opportunity for tailored advice.
